# Economic Entropy and the Cobb-Douglas Function: A Scientometric Analysis

**DOI:** 10.3390/e28050480

**Published:** 2026-04-22

**Authors:** Isabel Cristina Betancur-Hinestroza, Nini Johana Marín-Rodríguez, Francisco J. Caro-Lopera, Éver Alberto Velásquez Sierra

**Affiliations:** 1 Faculty of Economic Sciences, University of Medellin, Medellín 050026, Colombia; 2 Faculty of Engineering, University of Medellín, Medellín 050026, Colombia; njmarin@udemedellin.edu.co (N.J.M.-R.); fjcaro@udemedellin.edu.co (F.J.C.-L.); evelasquez@udemedellin.edu.co (É.A.V.S.)

**Keywords:** economic entropy, econophysics, Cobb-Douglas, utility function, production function, scientometric analysis

## Abstract

Economic entropy, as an emerging concept in econophysics, has gained increasing relevance in the analysis of complex systems characterized by uncertainty, nonlinearity, and out-of-equilibrium dynamics. However, its integration into conventional economic modeling—particularly in production functions such as the Cobb–Douglas function—remains fragmented and lacks systematic empirical validation. This study conducts a scientometric analysis of 345 Scopus-indexed documents (1973–2024) addressing the intersection between entropy, econophysics, and production functions, with the aim of mapping the intellectual structure of the field, characterizing its growth trends, identifying its core contributions, and highlighting its main research gaps. The results reveal that the field has experienced sustained growth since 2004, with a notable acceleration between 2020 and 2023, although it exhibits a fragmented authorship structure that does not conform to Lotka’s Law, suggesting that the field is still in a stage of scientific consolidation. The Cobb–Douglas function emerges as a niche topic within the econophysics literature, with limited integration between entropy-based approaches—informational, thermodynamic, and maximum entropy—and the empirical modeling of production. Furthermore, weak citation linkages between econophysics and conventional economics are observed, confirming the interdisciplinary fragmentation of the field. These findings provide a structured reference for researchers interested in advancing toward analytical frameworks that explicitly incorporate uncertainty, information, and physical constraints into economic analysis, thereby contributing to the development of econophysics as an integrative discipline.

## 1. Introduction

Econophysics has emerged as a rigorous interdisciplinary framework that draws on the mathematical and conceptual tools of statistical mechanics and thermodynamics to analyze complex economic phenomena [1]. Central to this framework is the concept of entropy—in its informational [2], thermodynamic [3], and maximum-entropy [4] formulations—which provides a principled means of quantifying uncertainty, disorder, and information in economic systems characterized by nonlinearity and out-of-equilibrium dynamics. These formulations are not merely analogical: Shannon entropy quantifies the informational content of economic signals and distributions; Boltzmann entropy connects macroeconomic states to the statistical weight of their microfoundations; and Jaynes’ maximum-entropy principle provides a normative framework for inference under incomplete information, all of which are directly relevant for economic modeling under uncertainty.

Classical economic models, however, have largely evolved independently of these developments. The Cobb–Douglas production function [5]—one of the most widely used models in economic theory and empirical research—was constructed under assumptions of constant returns to scale, factor substitutability, and equilibrium conditions that do not explicitly incorporate thermodynamic constraints, informational asymmetries, or nonlinear feedback processes [6]. While various authors have proposed generalizations of this function to address its empirical limitations, including approaches based on nonlinear growth dynamics and alternative scaling laws [7,8], its systematic integration with entropy-based principles remains underdeveloped. In particular, ref. [7] challenges the empirical validity of the classical formulation and proposes alternative frameworks grounded in logistic dynamics and empirical regularities such as Bowley’s law, highlighting the need to reconsider the foundations of production functions from broader theoretical perspectives. However, these developments have largely evolved in parallel with entropy-based approaches, revealing a conceptual disconnect in the literature. This represents a substantive gap at the intersection of econophysics and classical economic modeling.

Understanding the structure and evolution of research at this intersection is a necessary precondition for advancing the field. Scientometric and bibliometric methods provide a rigorous and reproducible approach to mapping the intellectual landscape of an emerging discipline: they allow researchers to identify dominant paradigms, detect thematic fragmentation, locate influential contributions, and uncover persistently neglected areas [9,10]. Indicators such as Lotka’s Law on author productivity [11] are particularly useful in this context, as they enable assessment of whether a scientific field has reached maturity or remains in an early stage of consolidation. Such analyses have been applied productively in related areas, including general econophysics [12] and the study of entropy across scientific domains [13]. However, to date, no study has conducted a targeted scientometric investigation specifically focused on the intersection of entropy, econophysics, and production and utility functions—particularly the Cobb-Douglas model. This gap is significant: without a structured map of the field’s intellectual landscape, it is difficult to identify where integration between entropy-based principles and economic modeling has genuinely advanced and where it remains conceptual or fragmentary.

The scope of this gap is further illustrated by the fact that foundational contributions at the boundary between thermodynamics and economic theory—such as the work of ref. [14] which establishes a formal correspondence between classical thermodynamics and general equilibrium theory—are not captured by bibliometric searches centered on econophysics terminology. Whether such works appear in a given corpus depends critically on the search strategy employed, and their potential absence is itself informative: it reflects the disciplinary fragmentation that prevents a unified vocabulary from consolidating across physics, mathematics, and economics.

This study addresses these gaps through a systematic bibliometric and scientometric analysis of 345 Scopus-indexed documents published between 1973 and 2024, using VOSviewer 1.6.19 [15] and the Bibliometrix package in R 4.3.2 [16] to map co-authorship networks, keyword co-occurrence structures, bibliographic coupling, and citation patterns. Unlike broader reviews of econophysics or entropy in the sciences, this analysis is explicitly focused on the role of entropy in economic production and utility modeling, making it possible to characterize not only what has been developed but—critically—what remains structurally underexplored or absent from the literature.

Three principal findings emerge. First, publication growth in this field has accelerated notably since 2004 and especially after 2017; however, the author productivity distribution does not conform to Lotka’s Law, suggesting that the field is still in a stage of scientific consolidation. Second, the Cobb-Douglas function appears as a niche theme within the econophysics literature, with limited empirical integration of entropy-based principles into production modeling. Third, there is a marked citational disconnect between contributions originating in econophysics and those developed within conventional economics—a pattern consistent with the resistance of mainstream economics to thermodynamic and informational approaches [17,18].

These findings have both theoretical and methodological implications. Theoretically, they highlight the underexplored potential of entropy—particularly maximum-entropy formulations in the tradition of ref. [4] and informational approaches derived from ref. [2]—to reframe production functions under conditions of uncertainty and resource constraints. Methodologically, they demonstrate that standard bibliometric searches based on econophysics terminology may systematically exclude relevant contributions from adjacent mathematical and economic studies [7,8,14], a limitation that is itself informative about the fragmented state of the field.

The remainder of this article is organized as follows. Section 2 describes the data sources, search strategy, and analytical methods. Section 3 presents the scientometric results across multiple dimensions of analysis. Section 4 discusses the implications of these findings and identifies directions for future interdisciplinary research.

## 2. Materials and Methods

This study conducted a systematic and targeted scientometric review to analyze the evolution of econophysics in relation to utility and production functions, focusing on contributions to the state of the art and advances at the frontier of knowledge. Scientometric analysis quantitatively examines academic studies to evaluate dynamics, structure, and scientific impact in a specific research area [9]. This approach enables the identification of research patterns, citation networks, co-authorship structures, and scientific productivity, providing insights into emerging trends and gaps.

Scopus served as the primary database for the systematic bibliometric corpus, given its extensive coverage of high-quality peer-reviewed literature and its robust indexing capabilities, making it ideal for a focused and reproducible bibliometric analysis. Google Scholar was consulted complementarily to verify coverage and to identify potentially relevant works not captured by the Scopus search strategy—particularly foundational contributions that do not employ econophysics terminology but establish formal bridges between thermodynamics and economic theory. However, given the well-documented reproducibility constraints of Google Scholar searches [19], all quantitative analyses reported in this study are based exclusively on the Scopus corpus of 345 documents.

The search terms were designed to capture the literature at the intersection of econophysics, entropy, and the Cobb-Douglas production function. The search strategy applied to Scopus was: TITLE-ABS-KEY(“econophysics”) OR TITLE-ABS-KEY(“entropy”) OR TITLE-ABS-KEY(“Entropy in economics”) OR TITLE-ABS-KEY(“First law of economics”) OR TITLE-ABS-KEY(“Thermodynamic formulation of economics”) OR TITLE-ABS-KEY(“Cobb-Douglas”) OR TITLE-ABS-KEY(“Production function”) OR TITLE-ABS-KEY(“Utility function”), combined with subject area filters: LIMIT-TO (SUBJAREA, “COMP”) OR LIMIT-TO(SUBJAREA, “MATH”) OR LIMIT-TO(SUBJAREA, “PHYS”) OR LIMIT-TO(SUBJAREA, “ECON”) OR LIMIT-TO(SUBJAREA, “SOCI”) OR LIMIT-TO(SUBJAREA, “DECI”) OR LIMIT-TO(SUBJAREA, “ENVI”) OR LIMIT-TO(SUBJAREA, “BUSI”) OR LIMIT-TO(SUBJAREA, “AGRI”) OR LIMIT-TO(SUBJAREA, “MULT”).

The use of OR operators rather than AND was deliberate. Given the fragmented and interdisciplinary nature of the field, a conjunctive search strategy would have excluded the majority of relevant documents, as few works at this intersection employ all three terms—entropy, econophysics, and production functions—simultaneously. This design choice reflects the structural fragmentation of the field itself: the absence of a unified vocabulary across physics, mathematics, and economics means that relevant contributions are distributed across terminologically distinct studies. The breadth of the OR-based search was compensated by the subsequent thematic filtering stage, in which studies outside the scope of econophysics, entropy, or the Cobb-Douglas function were excluded based on title, abstract, and keyword relevance.

The initial search yielded 345 documents spanning 1973 to 2024. Duplicates were removed using reference management software, followed by a manual review to ensure accuracy. The remaining documents were categorized based on their thematic alignment with the study objectives.

In addition to the systematic Scopus corpus, a small number of foundational references were incorporated through targeted expert review to provide theoretical context for the interpretation of results. These additional references—identified on the basis of their formal relevance to the thermodynamics–economics interface and cross-referenced from cited works within the corpus—include contributions that do not appear in the Scopus search due to terminological misalignment with econophysics vocabulary, such as the work of Smith and Foley [14] on the correspondence between classical thermodynamics and general equilibrium theory, and the work of Smirnov and Wang [7,8] on empirical generalizations of the Cobb-Douglas function. These references do not modify the quantitative results of the bibliometric analysis but inform the qualitative discussion of findings and research gaps, and their absence from the systematic corpus is itself treated as evidence of the interdisciplinary fragmentation documented in this study.

This study employed a structured bibliometric workflow beginning with document organization and preprocessing in R 4.3.2 using the Bibliometrix package [16]. Metadata standardization and keyword normalization were followed by network and clustering analyses using VOSviewer 1.6.19 [15]. The following parameter thresholds were applied to ensure analytical consistency and reproducibility: for co-authorship analysis, a minimum of two documents per author was required for inclusion in the network; for keyword co-occurrence analysis, only terms appearing in a minimum of three documents were retained; for co-citation analysis, a minimum citation threshold of five was applied; and network clustering was performed using VOSviewer’s 1.6.19 default resolution parameter (1.0) with a minimum cluster size of five items. Core techniques included co-authorship, co-citation, bibliographic coupling, keyword co-occurrence, and citation analysis. VOSviewer 1.6.19 was chosen for its interactive visualizations and scalability, while Bibliometrix was selected for its statistical robustness and integration with R-based analyses. These platforms provided an optimal balance between usability and analytical depth. The methodological design was informed by established scientometric mapping strategies [20], ensuring both analytical rigor and the ability to uncover the intellectual structure of the field.

The study was guided by the following research questions:

RQ1: Has research at the intersection of entropy, econophysics, and production functions grown significantly since 2004, and does its authorship structure reflect a mature scientific field?

RQ2: To what extent has the Cobb-Douglas function been empirically integrated with entropy-based principles in the existing literature?

RQ3: Is there evidence of citational integration between econophysics contributions and conventional economics in this domain, or does the literature exhibit structural fragmentation?

These research questions shaped the selection of methods and data interpretation, ensuring alignment between study objectives and conclusions.

## 3. Scienciometric Analysis

### 3.1. General Information

The study of economic entropy, emerging in the 1990s as an econophysics alternative to calculating production or utility functions, remains an underexplored field requiring further theoretical and applied validation. Scientometric analysis of 345 studies shows an average document age of 10.6 years and 29.37 citations, with publication growth increasing notably since 2004 at an annual rate of 4.29%, indicating rising but still limited academic engagement.

Approximately 74% of the documents are journal articles, followed by 20.8% conference papers. The field shows a moderate collaboration rate, with an average of 2.8 co-authors per document and 15.94% international co-authorship. Keyword diversity is high, with 2094 keywords plus and 1096 author keywords, suggesting thematic breadth. Despite a relatively small researcher network, collaboration is highly interconnected, as noted by [5] (Table 1).

#### 3.1.1. Annual Scientific Publication

**Figure 1:**
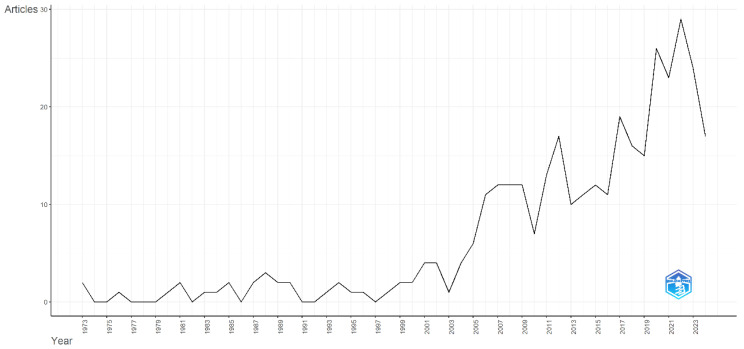
Annual scientific production. Source: Author’s own research using the Biliometrix tool with Scopus database.

The first publication related to econophysics, according to our search criteria, dates back to 1973 [21], analyzing entropy utility in spatial studies of shopping and work travel patterns in Detroit. Later, ref. [22] highlighted Palomba’s role in establishing the connection between physics and economics in Economic Physics, alongside ref. [23], who linked thermodynamics and economic processes. Academic production in econophysics, particularly in production and utility functions, has surged since 2004, with 87.83% of the 345 published documents concentrated in the last 20 years. Since 2017, publication rates have increased significantly, averaging 20 per year, with the highest average (25.5 per year) recorded between 2020 and 2023 (see Figure 1).

Some of the topics addressed in these studies prior to 2004 included entropy [6,24,25], utility functions [26,27,28], mathematical models [29,30,31], and decision-making [28,32,33], among others. It is notable that these publications accounted for almost 13% of the total. Subsequently, from 2004 onwards, the focus on entropy continued [34,35,36,37], along with studies on utility functions [38,39,40], decision-making [36,41,42]. Additional topics explored included information theory [36,41,43,44], relative entropy [41,45,46,47,48] and various studies on land use in China [49,50,51].

The scientometric analysis identified production functions as a key topic, with themes such as technical efficiency, productivity evaluation, and specific production models explored in both theoretical and applied contexts [12,52,53,54,55,56]. However, practical applications remain limited, highlighting the need for further research to strengthen econophysics’ role in economic analysis. Empirical studies have applied entropy to estimate production functions, including ref. [25] using generalized maximum entropy (GME) with simulated data, ref. [52] analyzing FAO data from Mexican farms, and ref. [57] estimating production functions in Spanish regions. Ref. [58] employed Shannon entropy, while ref. [59] used entropy to develop a pollution indicator. Theoretical contributions, such as those by refs. [12,60] compare the Cobb-Douglas function with entropy, suggesting that economic entropy could provide superior insights. These findings underscore the need for further investigation to enhance economic modeling through entropy-based approaches.

In the area of Cobb-Douglas production functions and entropy, there have been few recent publications. Given the potential significance of this topic, it would be valuable to encourage further research, particularly with empirical approaches. This would aid in validating and advancing the existing theoretical constructions.

#### 3.1.2. Most Relevant Journals

**Figure 2:**
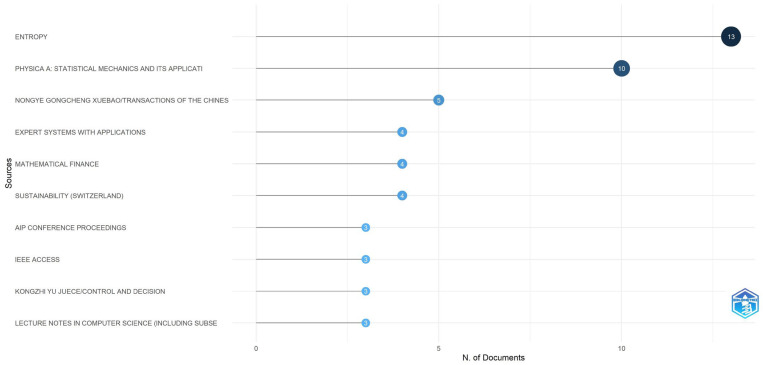
Most relevant Journals. Source: Author’s own research using the Biliometrix tool with Scopus database.

Figure 2 highlights the top scientific journals publishing on econophysics from the perspective of production and utility functions. Entropy leads in publications, followed by Physica A and other journals with fewer contributions. Around 16.8% of articles appear in the top 10 journals, indicating a broad distribution across various sources.

Specifically, Entropy focuses on topics related to information theory, entropy, and thermodynamics, exploring the use of entropy across various disciplines, including the social sciences. It covers subjects such as maximum entropy and information theory [47,61], statistical models and methods [52,62] and applications in economics and finance [61,63]. Although Physica A primarily publishes scientific articles focused on statistical mechanics and its applications, it also addresses a wide range of topics, including econophysics [55,64,65], modeling of economic behavior and distributions [66,67], and the questioning and revision of classical economic theories [65,68]. Additionally, Nongye Gongcheng Xuebao, also known as Transactions of the Chinese Society of Agricultural Engineering, has a specific focus on agricultural engineering, mainly in China. According to the search criteria for this research, it has been published on topics such as land use evaluation and planning [69,70,71,72]. Expert Systems with Applications specializes in research on artificial intelligence and its applications in various areas, including the social sciences. Based on the search criteria, this journal has published on topics related to the optimization and improvement of data-driven strategies, the use of utility functions, and probabilities in decision-making, among others [73,74]. Finally, Mathematical Finance specializes in applying mathematical techniques and models to financial problems. The journal covers topics such as risk management, market models, and portfolio theories, among others. According to the search criteria, articles in this journal use entropy to optimize and improve decision-making and analysis strategies [24], as well as apply the utility function within financial contexts, such as the evaluation and maximization of expected benefits [41,75,76]. It is important to highlight that the first applications of econophysics were conducted in the financial domain, contributing to the development of innovative models for derivative valuation and market trend prediction [1,77,78].

#### 3.1.3. Most Cited Sources

The data presented in Table 2 shows the top 10 journals or books in the field of econophysics from the perspective of production functions and utility, utilizing metrics such as the h-index, g-index, total citations, number of publications, and the Scimago ranking. These metrics help evaluate both the productivity and impact of the journals, providing a comprehensive perspective on their standing in the scientific community. The results highlight outstanding performance in these metrics and their contribution to this field.

Regarding the most cited sources, we find that the majority also have the highest citation counts, indicating a close correlation with the previous analysis. Notably, the journals Entropy and Physica A have the highest h-index on this list, with a value of 7, indicating that both journals have at least 7 articles cited a minimum of 7 times each. Additionally, they have the highest g-index, 11 for Entropy and 10 for Physica A, suggesting that some of their articles are highly cited. Furthermore, the journals Nongye Gongcheng Xuebao, Mathematical Finance, and Sustainability have lower h and g indices, but Mathematical Finance has an exceptional number of citations at 608, with only 4 publications, indicating that these publications are highly influential and frequently cited, reflecting a significant impact.

In terms of total citations and the number of publications for the remaining journals, we find that Entropy and Physica A have a balanced number of citations, with 134 and 114, respectively, which, in relation to their number of publications, 13 and 10, respectively, indicates a strong impact and recognition within their fields. Although IEEE Access and Operations Research have a moderate number of publications, they have a substantial total number of citations (97 and 228, respectively), suggesting a good level of impact. While Econophysics and Sociophysics: Trends and Perspectives is a book rather than a traditional journal, it has a significant number of citations (271, the third highest), and when calculating the average number of citations, it achieves the second-highest value (135.5), after Mathematical Finance (152 average citations). However, it is important to note that the book delves deeper into the field of econophysics from the perspective of production functions and utility, indicating that this publication is of great relevance as a reference in this field of study.

Regarding the Scimago categories, although Mathematical Finance, Sustainability, IEEE Access, and Operations Research are classified in the highest quartile Q1, they do not have as many publications as Entropy, Physica A, and Nongye Gongcheng Xuebao, which, despite being in a slightly lower quartile Q2, have more publications as well as higher g and h impact indices. Both categories are considered and prestigious.

The metrics mentioned indicate that the journal Mathematical Finance has the highest total number of citations with only four publications and solid g and h indices of 4 each, in addition to its Q1 category in Scimago. However, according to the specific theme of this bibliometric analysis, other sources such as the journal Entropy and the book Econophysics and Sociophysics: Trends and Perspectives offer publications with significant approaches and foundational insights that serve as a starting point for the specific field of study in econophysics from the perspective of production functions and utility.

### 3.2. Core Authors

#### 3.2.1. Authors’ Productivity

**Figure 3:**
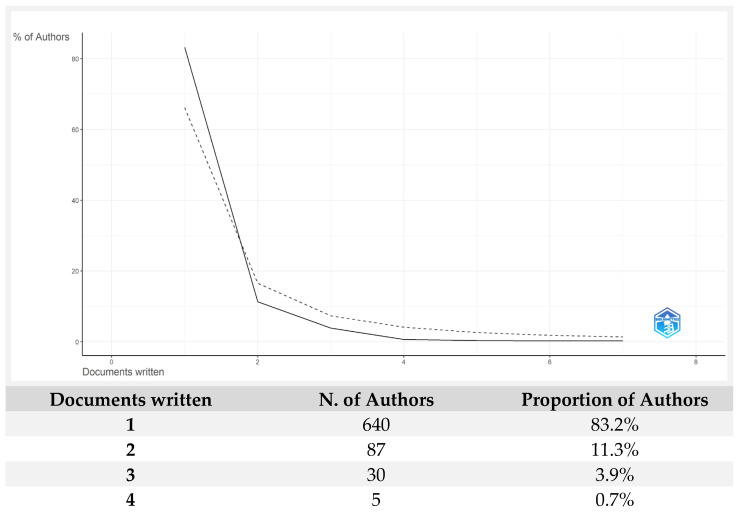
The author’s productivity. Source: Author’s own research using the Biliometrix tool.

Lotka’s Law [11] identifies and characterizes researchers who exhibit a higher frequency of publications in a specific area of knowledge. Figure 3 presents the results of the articles and their distribution according to Lotka’s Law on econophysics from the perspective of production functions and utility. In this study, the results show a Lotka index where 83.2% of the authors would write one article, 11.3% would write two, 3.9% would write three, and only 0.7% would write four. These data suggest that topics related to econophysics from the perspective of production functions and utility currently do not conform to Lotka’s Law. The dashed line in the figure represents the graph that should align with Lotka’s Law.

This implies that, instead of observing the typical pattern where most researchers produce only a small number of publications while a small group of researchers produces a large number of publications, a different distribution is emerging. This may be due to a lower concentration of publications among a few authors, the fact that this field of study is still in development, or other contributing factors.

#### 3.2.2. Authors’ Productivity over Time and Most Relevant Authors and Authors’ Impact

**Figure 4:**
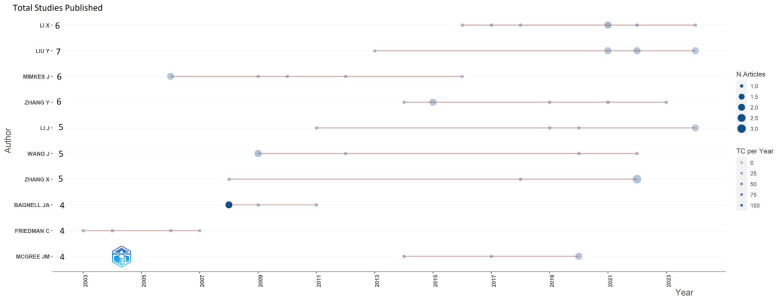
The author’s productivity over time. Source: Author’s own research using the Biliometrix tool.

Figure 4 presents the most prominent authors who have made significant contributions to research in econophysics from the perspective of production functions and utility, as indicated by the number of studies published. In this figure, the size of the bubbles represents the number of publications, while the intensity of the color reflects citations per year, calculated based on the total citations received from the time of publication up to the year 2024.

Among the most notable authors in this field is J. Mimkes, who was particularly active in terms of publications around 2006. Although he continued to publish steadily in the following years, no further publications are recorded after 2016. Mimkes is known for his work on integrating entropy and thermodynamics into economic analysis, providing a unique perspective on the interaction between physics and economics [55,79]. Similarly, Y. Zhang and J. Wang exhibited a high number of publications at the beginning of their careers, though their output decreased toward 2023. The former has focused on applying physical models related to the application of entropy and advanced statistical methods to fault diagnosis and complex systems analysis [80].

On the other hand, the 2008 publication by J.A. Bangell, which developed a probabilistic approach based on the principle of maximum entropy to tackle complex machine learning problems, received the highest number of citations according to bibliometric results obtained. However, publications from this author are only observed until 2011. In contrast, X. Li, Y. Liu, and J. Li have maintained a steady output in recent years, with special mention to Y. Liu, who has published eight papers from 2013 to 2024. Nevertheless, their work focuses more on contexts related to energy, health sciences, and the environment, using entropy principles and utility.

When analyzing the number of publications and the number of authors per article, although X. Li and Y. Liu have the highest number of articles, their fractionalized article scores are 1.8 and 2.57, respectively, suggesting significant collaboration with other authors. However, this also indicates that their individual contributions may be smaller in each publication. According to these results, J. Mimkes, with his 6 reported articles, has a fractionalized article score of 6, indicating that he is the sole author and that his contribution is substantial relative to the number of publications made, and the highest according to the data reported in Table 3.

Recent publications highlight the need for greater theoretical and practical contributions in econophysics, with an emphasis on production and utility functions. Authors with higher fractional scores contribute more directly, while lower scores indicate greater collaboration. This suggests that J. Mimkes plays a significant role in the field.

#### 3.2.3. Most Relevant Author’s Affiliation

**Figure 5:**
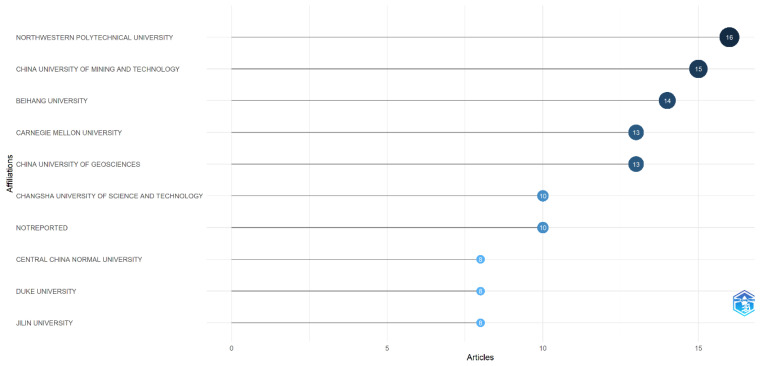
Most relevant author’s affiliation. Source: Author’s own research using the Biliometrix tool.

Figure 5 highlights the leading institutions that have made significant contributions to research on econophysics with a focus on production functions and utility. This ranking is based on the number of published studies, with the following five institutions emerging as the top contributors: (i) Northwestern Polytechnical University with 16 published articles, (ii) China University of Mining and Technology with 15 articles, (iii) Beihang University with 14 published articles, (iv) Carnegie Mellon University with 13 articles, and (v) China University of Geosciences, also with 13 articles.

Northwestern Polytechnical University and China University of Mining and Technology report the highest number of publications in this field of study, which may indicate their specialization in this area and their significant contributions. This outcome could also be related to the presence of research groups and collaborations among authors.

#### 3.2.4. Author’s Country Analysis

**Figure 6:**
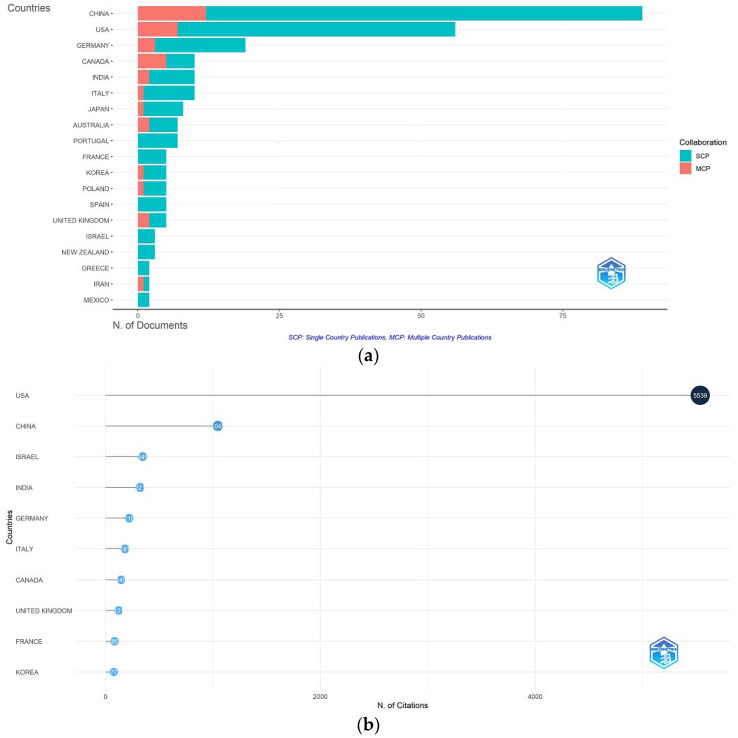
(**a**) Corresponding author’s countries. (**b**) Most cited countries.

Figure 6a highlights the leading countries in econophysics research on production and utility functions, with China, the United States, and Germany standing out. In China, key institutions include China Northwestern Polytechnical University and China University of Mining and Technology, while Carnegie Mellon University leads in the U.S. The figure also shows that most publications from these countries are collaborative efforts; in China, 77 out of 89 articles involved international co-authors, while in the U.S., 49 out of 56 were produced through collaborations (Table 4).

Figure 6b reveals that, while China, the United States, and Germany lead in econophysics publications on production and utility functions, their global impact varies significantly. The United States ranks highest in total citations (5539), followed by China (1045), Israel (345), and India (321). Despite fewer publications, Israel has the highest average citations per article (115), surpassing the U.S. (98.9) and Colombia (50), indicating a strong research impact. While international collaboration enriches scientific quality, factors such as content relevance and institutional reputation also shape academic influence, with the U.S. leading in overall impact despite fewer international co-authorships than China.

### 3.3. Core Studies

Citation analysis is a common method to measure research impact [81,82], assessing the influence and relevance of academic work [83]. It provides a quantitative measure of recognition, showing how research is disseminated, accepted, and influences other fields, while also identifying emerging trends and guiding future studies. Table 5 displays the most significant studies measured by total citations. These topics focus on the use of entropy and utility maximization in various contexts such as finance [24,41,45], decision theory [36,84] and robotics [85]. There is also a strong emphasis on mathematical and computational applications to solve practical problems in economics [12], finances [24,41,45,75] and dynamic systems [86,87].

Ref. [35] explores strictly proper scoring rules, essential tools in statistical forecasting that ensure forecasters report true probabilistic beliefs, enhancing prediction accuracy and decision-making. Ref. [40] applies the maximum entropy principle to inverse reinforcement learning, refining artificial intelligence models that infer agent goals from observed behavior, thus improving adaptability in AI systems. Ref. [88] develops an axiomatic theory for fair resource allocation in networks, providing guidelines for equitable and efficient distribution in fields like telecommunications. Ref. [12] examines econophysics and sociophysics, demonstrating how statistical physics methods can analyze economic and social dynamics, highlighting their role in wealth distribution, stock markets, and collective behavior modeling. This interdisciplinary approach fosters collaboration among physicists, economists, and sociologists, promoting methodological innovation in complex system analysis.

Refs. [24,28,45] contribute to financial decision-making through risk, utility, and optimization studies. Ref. [24] integrates utility maximization and entropy in financial pricing, providing a flexible asset valuation framework under market constraints. Ref. [28] extends the optimized certainty equivalent as a convex risk measure, allowing more comprehensive risk assessment. Ref. [45] focuses on dynamic revenue management, optimizing pricing policies under uncertainty using relative entropy to address demand model imperfections. Their work strengthens pricing strategies in uncertain markets, enabling businesses to make more robust financial decisions.

#### 3.3.1. Co-Occurrence of Keywords Analysis

Keyword co-occurrence analysis identifies relationships and patterns among concepts in large text volumes, enabling thematic connections, improving scientific searches, and detecting emerging trends. Ref. [90] states that these connections can form an ontology to structure knowledge, but emphasizes the importance of preserving researchers’ interpretative ability in the analysis.

**Figure 7:**
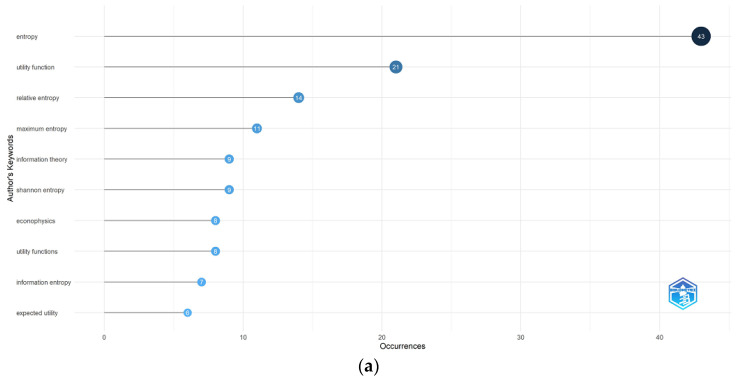

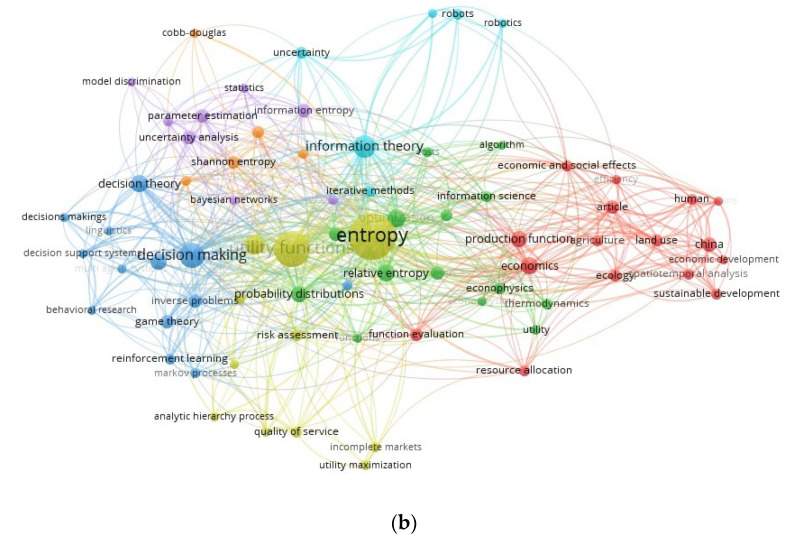
(**a**). Most Frequent Words. (**b**). Concurrence Analysis.

Figure 7a,b shows that entropy is the most predominant keyword in research on econophysics and production or utility functions, followed by utility functions, decision making, information theory, and economics. There is an interconnection between utility functions and microeconomic concepts such as game theory and risk assessment, while information theory is linked to decision making, economics, and uncertainty analysis. Additionally, production functions are associated with maximum entropy, agriculture, and land use. Peripheral topics such as robotics, Cobb–Douglas, and incomplete markets remain underexplored, highlighting the need to expand research and establish new connections in these areas.

#### 3.3.2. Thematic Map

**Figure 8:**
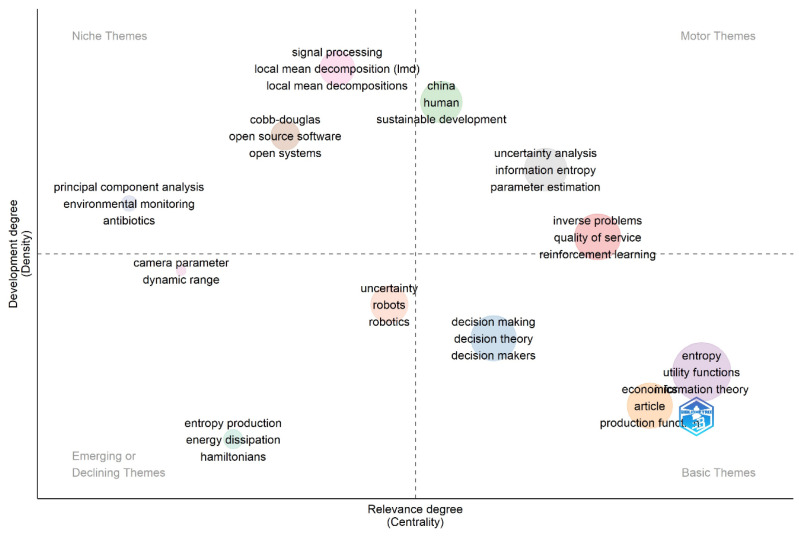
Thematic map using keywords plus. Source: Author’s own research using Biliometrix.

Figure 8 presents a thematic map that organizes research topics based on their importance and development [91]. This approach helps identify emerging and fundamental trends, facilitating connections between study areas and promoting interdisciplinary work. It also measures each topic’s contribution to the research field [92].

The map classifies topics into four quadrants: motor themes (highly developed and relevant), niche themes (specialized but less impactful), emerging themes (underdeveloped or declining), and basic themes (high growth potential). This classification guides future research and highlights the most productive and influential areas.

The results from Figure 8 indicate that the Motor Themes quadrant lacks topics with high centrality, but service quality, reinforcement learning, uncertainty analysis, information entropy, and parameter estimation stand out for their development. Sustainable development and applications in China require greater centrality, suggesting the need for further research. Although these topics are advanced, they could benefit from increased academic attention.

Within the Niche Themes quadrant, several areas—such as principal component analysis, environmental monitoring, Cobb-Douglas, open-source software, open systems, signal processing, and local mean decomposition—are identified as well-developed yet isolated fields [93]. To increase their relevance in econophysics and utility or production functions, researchers should explore applications like using principal component analysis for financial data dimensionality reduction, integrating ecological variables into utility modeling, and empirically expanding Cobb-Douglas functions to incorporate changes in technology and energy use.

Previous works offer valuable precedents: ref. [51] examined spatial-temporal dynamics in “production-living-ecological” systems; refs. [94,95] studied environmental risks in pharmaceutical and agricultural systems. In the context of production modeling, refs. [58,96,97,98] developed quantitative tools to assess code complexity. Ref. [99] applied Dempster-Shafer theory and the Maximum Entropy Principle to supplier selection under uncertainty. Ref. [100] used thermodynamic integrals to analyze macroeconomic phenomena, challenging neoclassical assumptions and advancing stochastic approaches in financial markets.

In the Emerging Themes quadrant, underexplored areas such as uncertainty, robotics, entropy production, and Hamiltonians could gain traction by aligning with more established topics like decision-making, entropy, and production functions. Doing so could foster interdisciplinary integration and broaden their theoretical and applied relevance.

The Basic Themes quadrant includes foundational yet dynamic topics such as entropy, utility functions, production functions, information theory, and decision theory. These themes offer high development potential, indicating a pressing need for further research to maximize their conceptual and practical contributions [101].

#### 3.3.3. Theories, Contributions and Trends

Econophysics, through tools like entropy, enhances the understanding of uncertainty and disorder in economic systems. Economic entropy helps measure dispersion and efficiency in resource allocation, essential for both production and utility functions. In the Cobb-Douglas production function, entropy facilitates production calculations by linking thermodynamic and economic variables [12]. Likewise, in the utility function, entropy integrates uncertainty into consumer preferences, capturing behavioral variability [102]. The following section outlines key theories, contributions, and trends.

The relationship between econophysics and production or utility functions can be examined through three major theoretical frameworks. First, information theory and entropy offer tools to quantify uncertainty and complexity in resource allocation. Unlike traditional models such as Cobb-Douglas, entropy-based approaches allow for modeling variability and inefficiency in production. Shannon entropy helps characterize unpredictability, while Jaynes’ principle of maximum entropy enables inference under limited information, both in production efficiency and consumer preference modeling [2,4,103].

Second, thermodynamic and statistical mechanics theories model economic systems analogously to physical systems. The second law of thermodynamics is applied to understand long-term inefficiencies in production and consumption patterns. This framework suggests that marginal utility behaves entropically, diminishing as consumption increases, and that agents adjust preferences based on constraints and resource availability [4,104].

Third, mathematical finance and stochastic models integrate randomness into production and utility analysis. These models capture volatility and exogenous shocks through stochastic processes, reflecting real-world fluctuations in inputs and outputs. In finance, such models are used to assess asset values and risks, and they can be extended to utility theory to evaluate decisions under uncertainty [24,105,106,107]. Together, these lenses enrich the analysis of complex economic behavior beyond deterministic assumptions.

The contributions from econophysics in relation to production and utility functions can be classified into:

- The relationship between economic growth and entropy: In the economic field, entropy has been applied to understand the evolution of economies through endogenous growth, highlighting the role of knowledge and innovation in capital accumulation and long-term sustainable development [54,108,109].

Trends in econophysics in relation to production and utility functions can be classified into:

- Optimization in decision-making in complex environments: Methods based on entropy and cumulative prospect theory have been crucial for improving decision-making in multi-criteria scenarios. These approaches have enabled the application of robust models in supplier selection, network security evaluation, and classification of multi-attribute problems, optimizing decision-making accuracy and efficiency [87,110,111].
- Utility and efficiency analysis through entropy: Entropy has been employed in evaluating utility and efficiency in various contexts, from agricultural production to maritime surveillance. These models optimize resource allocation and improve technical efficiency while providing a framework for analyzing decision-making under uncertainty and risk scenarios [112,113,114].
- Applications of artificial intelligence and optimization in distributed systems: Advances in artificial intelligence and machine learning, together with entropy, have facilitated optimization in distributed systems, particularly in cooperative information search and efficient data transmission. These approaches have improved the detection of multiple targets in dynamic environments, with applications in fields such as robotics and image transmission [73,115,116].

### 3.4. Citation Network Analysis

#### 3.4.1. Bibliography Coupling

Bibliographic coupling is a bibliometric method that identifies thematic relationships between scientific articles based on shared references. When multiple documents cite the same sources, they indicate a common knowledge area, revealing the structure and theoretical foundations of a research field. This method is essential in citation network analysis for mapping academic literature [117].

**Figure 9:**
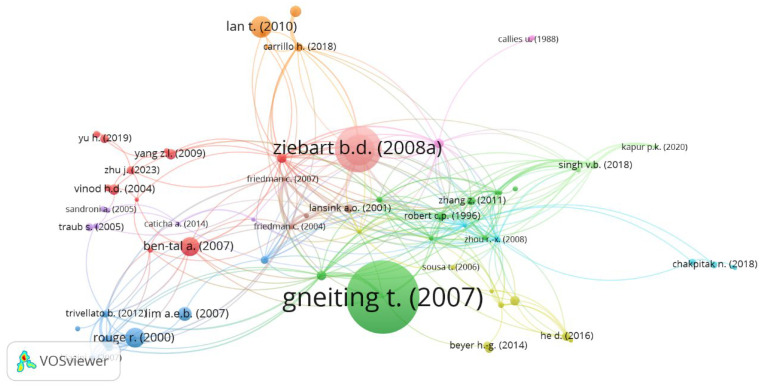
Bibliographic Coupling [24,35,38,40,41,42,45,48,58,62,65,84,88,97,118,119,120]. Source: Author’s own research using VOSviewer 1.6.19.

The results align with Table 5, which lists the 15 most cited articles on econophysics and production or utility functions. Ref. [35] explores decision-making under uncertainty, linking entropy to improving probabilistic forecasts and parameter estimation in probabilistic models. Similarly, ref. [41] examine divergence, relating entropy to coherent risk measures, focusing on information theory and convex duality (Figure 9).

This analysis highlights how entropy is applied to address uncertainty and fairness in economic and behavioral modeling. Ref. [40] uses the maximum entropy principle to resolve underdetermination in observed behaviors, selecting policies that distribute probabilities most evenly across actions. Ref. [88] introduces five axioms for fairness in resource allocation, identifying entropy as a measure that aligns with these criteria. Ref. [24] integrates entropy into expected utility maximization, enhancing pricing models by accounting for uncertainty and market volatility.

#### 3.4.2. Co-Authorship Analysis

This analysis explores author collaboration networks across scientific publications to identify patterns of co-authorship and interdisciplinary engagement. By mapping these networks, it becomes possible to detect emerging collaboration trends, highlight leading researchers within specific fields, and understand the formation of intellectual schools of thought. The approach also facilitates the identification of interdisciplinary linkages and provides metrics for assessing institutional cooperation [121].

**Figure 10:**
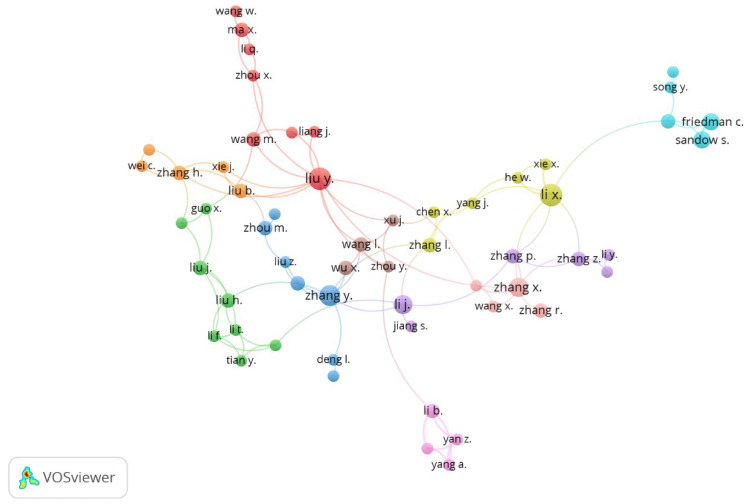
Co-Authorship. Source: Author’s own research using VOSviewer 1.6.19.

Figure 10 illustrates a network of authors with at least two publications and one citation, organized into five principal clusters, each reflecting specific research themes and collaboration patterns. The red cluster, led by Liu Y., Wang M., Li Q., and Zhou X., is highly central and well-connected, indicating strong interdisciplinary collaboration. Their work emphasizes network analysis, multi-criteria decision-making, and territorial development [70,89,122]. 

The green cluster, headed by Liu H., Liu J., and Guo X., shows strong internal cohesion but fewer external ties, suggesting a specialized focus. Their research includes network optimization, entropy-based analysis, rural territorial planning, and pricing strategies [116,123,124], with a clear interest in system efficiency and uncertainty management via entropy.

In the yellow cluster, Li X. and He W. engage in cross-disciplinary work on mechanical fault diagnosis, Bayesian modeling, and spatial land planning through functional zoning [51,125,126]. This integration of advanced signal processing with sustainable territorial development reflects an emphasis on optimizing complex systems through technological innovation.

The blue cluster, composed of Zhang Y., Zhou M., and Liu Z., concentrates on mechanical systems, particularly bearing fault diagnosis and decision-making under uncertainty, with entropy as a core analytical tool [36,127]. Their work remains technically specialized, with limited interdisciplinary engagement.

The light blue cluster, a smaller and more isolated group led by Friedman C., Sandow S., and Song Y., centers on expected utility theory, decision analysis, entropy, and environmental modeling [47,118,119]. Their contributions merge mathematical and probabilistic theories with applied data modeling in ecological and land use contexts.

Overall, Figure 10 reveals a diverse and structured network of scientific collaborations. While some groups (e.g., red and yellow) exhibit strong interdisciplinary links, others maintain a narrow technical scope. The authors’ roles in each cluster shape the network’s intellectual structure, spanning topics from entropy and decision theory to sustainable development and mechanical optimization.

## 4. Discussion

This study set out to map the intellectual structure of research at the intersection of entropy, econophysics, and production and utility functions—an area that, despite growing interest, had received no prior targeted scientometric treatment. Three research questions guided the analysis, and the following discussion interprets the principal findings in light of each.

The sustained acceleration in publication output since 2004—particularly after 2017—confirms that this intersection constitutes an active and growing area of scientific inquiry, consistent with broader trends in econophysics since the foundational contributions of Mantegna and Stanley [1]. However, the finding that author productivity does not conform to Lotka’s Law warrants careful interpretation. Lotka’s Law predicts that in a mature field, a small number of highly productive authors concentrate a disproportionate share of total output [11]; the deviation observed here—where 83.2% of authors contributed a single document—indicates that the field has not yet developed the concentrated intellectual leadership that characterizes established disciplines. This is consistent with the observation that progress in this area is occurring largely outside the mainstream of either physics or economics [17,18], without the institutional anchoring—specialized journals, consolidated research groups—that typically drives the concentration of scientific productivity Lotka describes. The distribution of 74% of documents across 265 different sources implies that the field is growing, but in a dispersed manner.

The central finding—that the Cobb-Douglas function appears as a niche theme within the econophysics literature, with limited empirical integration of entropy-based principles into production modeling—is not a peripheral observation but the substantive contribution of this study. The Cobb-Douglas function was chosen as the focal point and starting benchmark for two reasons. First, it remains the predominant functional form for production functions in applied economic work, serving as what leading production economists have called the ‘gold standard’ of neoclassical production theory for nearly a century [128]. Second, its mathematical structure—a power-law relationship between inputs and output of the form Y = AK^α^L^β^—establishes a formal bridge with entropy-based approaches: power-law distributions are a signature of maximum-entropy processes under scale-invariance constraints [4], making Cobb-Douglas the natural starting point for any integration of entropic principles into production modeling.

This absence is not uniform across entropy formulations. Informational approaches—particularly Shannon entropy and maximum entropy methods—have found more traction than thermodynamic approaches, like applications to production function estimation [52], which is documented in the corpus, but remain largely theoretical or simulation-based. Thermodynamic approaches, as attempted by refs. [55,65], remain the least developed empirically despite being conceptually the most ambitious.

This result must be read alongside recent work by refs. [7,8], who have demonstrated that empirical data do not fit the classical Cobb-Douglas formulation well and have proposed alternative frameworks grounded in logistic dynamics and Bowley’s law. The fact that these generalizations have emerged independently of entropy-based approaches—with limited cross-citation between the two lines of research—is itself a finding, as it confirms that the reconsideration of the foundations of production functions is occurring simultaneously in multiple disciplinary spaces without the cross-fertilization that would accelerate progress.

The marked citational disconnect between econophysics and conventional economics—confirmed by the co-citation and bibliographic coupling analyses—is a consequential finding of this study. This is not a new observation, refs. [17,18] have documented the resistance of mainstream economics journals to thermodynamic and informational approaches—a resistance historically anticipated by Samuelson’s explicit dismissal of entropy analogies in his 1970 Nobel lecture [129]. Likewise, the concentration of publications in Entropy and Physica A means that entropy-based approaches to production modeling are largely invisible to economists who work daily with the Cobb-Douglas function or other traditional economic frameworks, while generalizations of that function developed within economics—such as those of refs. [7,8]—do not appear in econophysics searches due to terminological misalignment.

The work of ref. [14]—which establishes a formal correspondence between classical thermodynamics and general equilibrium theory—does not appear in Scopus searches centered on econophysics terminology. Its absence from the systematic corpus, and its incorporation here through targeted expert review, is itself a demonstration of the fragmentation this study documents, and points to a broader methodological lesson: scientometric analyses of interdisciplinary fields must complement automated searches with expert-guided identification of contributions that transcend disciplinary boundaries.

Three directions emerge for future research. First, empirical validation: maximum entropy estimation methods should be applied to real, disaggregated production data and systematically contrasted with classical Cobb-Douglas specifications—the most urgent gap identified in this corpus [120]. Second, bridge-building between disciplines: overcoming terminological and citational fragmentation requires collaborative research programs and publication venues positioned at the disciplinary interface, as Entropy has demonstrated. Third, systematic comparison of entropy formulations: Shannon, Boltzmann, and Jaynes entropy have been applied to economic modeling in distinct and largely unconnected ways; identifying under what empirical conditions each is most appropriate could provide a more unifying theoretical framework.

## 5. Conclusions

This study presents the first targeted scientometric analysis of research at the intersection of entropy, econophysics, and production and utility functions, based on 345 Scopus-indexed documents published between 1973 and 2024. The analysis was guided by three research questions whose answers, taken together, offer a structured diagnosis of the field’s current state and its principal challenges.

With respect to the growth and maturity of the field (RQ1), the results confirm a sustained acceleration in publication output since 2004, with an average annual growth rate of 4.29% and a notable intensification between 2020 and 2023. The journals Entropy and Physica A have emerged as the primary publication platforms, concentrating 16.8% of total output—a finding that is itself significant, as it confirms that this research program is institutionally anchored in physics and information science rather than in economics. However, the author productivity distribution does not conform to Lotka’s Law: 83.2% of authors contributed a single document, indicating that the field has not yet developed the concentrated intellectual leadership that characterizes mature scientific disciplines.

With respect to the integration of entropy with the Cobb-Douglas function (RQ2), the central finding is that this integration remains largely absent from the empirical literature. The Cobb-Douglas function—chosen as the focal point of this analysis because it is one of the most widely used empirical specifications in macroeconomic and growth modeling—emerges as a niche theme within the econophysics corpus. Informational approaches, particularly maximum entropy methods, have found more traction than thermodynamic approaches, but both remain primarily theoretical and rely on simulated rather than real production data. This is not a minor gap: the Cobb-Douglas function is the reference point against which any entropy-based production theory must ultimately be evaluated, and the absence of systematic empirical validation may mean that the theoretical case for entropy-based production modeling, however compelling, remains unsubstantiated at the level of applied economics.

With respect to citational fragmentation between econophysics and conventional economics (RQ3), the co-citation and bibliographic coupling analyses reveal a marked disconnect between the two studies. Entropy-based contributions to production modeling are largely invisible to economists who work daily with the Cobb-Douglas function, while recent generalizations of that function—such as those proposed by refs. [7,8]—do not appear in econophysics searches due to terminological misalignment. This fragmentation is not merely an inconvenience: it may mean that parallel lines of research are independently questioning the foundations of classical production theory without the cross-fertilization that would accelerate progress in either direction. The historical resistance of mainstream economics to entropy-based reasoning—documented by refs. [17,18]—has contributed to this institutional separation, which this study now documents empirically at the level of citation networks.

Taken together, these three findings yield a coherent diagnosis: the field of economic entropy as applied to production and utility functions is growing but fragmented, theoretically developed but empirically underdeveloped, and institutionally anchored in physics while remaining largely disconnected from the economics literature it seeks to inform. Three priorities emerge for future research. First, empirical validation: maximum entropy estimation methods should be applied to real, disaggregated production data and systematically contrasted with classical Cobb-Douglas specifications and other traditional economic functions—the most urgent gap identified in this corpus. Second, bridge-building between disciplines: overcoming terminological and citational fragmentation requires collaborative research programs and publication venues positioned at the disciplinary interface. Third, systematic comparison of entropy formulations: Shannon, Boltzmann, and Jaynes entropy have been applied to economic modeling in distinct and largely unconnected ways; identifying under what empirical conditions each is most appropriate would provide the unifying theoretical framework the field currently lacks.

## Figures and Tables

**Table 1:** Summary of the studies.

Description	Results	Description	Results
MAIN INFORMATION ABOUT DATA		Authors of single-authored docs	61
Timespan	1973:2024	AUTHORS COLLABORATION	
Sources (Journals, Books, etc.)	265	Single-authored docs	72
Documents	345	Co-Authors per Doc	2.8
Annual Growth Rate %	4.29	International co-authorships %	15.94
Document Average Age	10.6	DOCUMENT TYPES	
Average citations per doc	29.37	article	256
References	11,434	book	2
DOCUMENT CONTENTS		book chapter	7
Keywords Plus (ID)	2094	conference paper	72
Author’s Keywords (DE)	1096	conference review	2
AUTHORS		retracted	1
Authors	769	review	5

Source: Author’s own research using the Biliometrix tool.

**Table 2:** Top highly cited sources.

Element	h Index	g Index	Total Citations	Number Publications	Top Category Scimago
Entropy	7	11	134	13	Q2
Physica A: Statistical Mechanics And Its Applications	7	10	114	10	Q2
Nongye Gongcheng Xuebao/Transactions Of The Chinese Society of Agricultural Engineering	5	5	49	5	Q2
Mathematical Finance	4	4	608	4	Q1
Sustainability (Switzerland)	4	4	74	4	Q1
Ieee Access	3	3	97	3	Q1
Operations Research	3	3	228	3	Q1
Autonomous Robots	2	2	59	2	Q1
Econometric Reviews	2	2	12	2	Q1
Econophysics And Sociophysics: Trends And Perspectives	2	2	271	2	Book

Source: Author’s own research using the Biliometrix tool.

**Table 3:** Most Cited Authors.

Authors	Articles	Articles Fractionalized
Li X	7	1.80
Liu Y	7	2.57
Mimkes J	6	6.00
Zhang Y	6	1.37
Li J	5	1.38
Wang J	5	2.08
Zhang X	5	1.57
Bagnell Ja	4	1.08
Friedman C	4	1.83
Mcgree Jm	4	2.00

Source: Author’s own research using the Biliometrix tool.

**Table 4:** Most Cited Countries.

Country	TC	Average Article Citations
Israel	345	115.00
USA	5539	98.90
Colombia	50	50.00
Austria	38	38.00
India	321	32.10
United Kingdom	121	24.20
Italy	181	18.10
Thailand	35	17.50
France	85	17.00
Korea	77	15.40

Source: Author’s own research using the Biliometrix tool.

**Table 5:** Top 15 Most globally cited studies.

No.	Authors	Source	Year	Total Citation	Type
1	Gneiting & Raftery (2007) [35]	Strictly proper scoring rules, prediction, and estimation	2007	3158	Article
2	Ziebart et al. (2008) [40]	Maximum entropy inverse reinforcement learning	2008	1286	Conference paper
	Ziebart et al.(2008) [40]	Maximum Entropy Inverse Reinforcement Learning	2008	563	Conference paper
3	Lan T et al. (2010) [88]	An axiomatic theory of fairness in network resource allocation	2010	279	Conference paper
4	Chakrabarti B.K. (2006) [12]	Econophysics and Sociophysics: Trends and Perspectives	2006	258	Book
5	Rouge & El Karoui (2000) [24]	Pricing via utility maximization and entropy	2000	244	Article
6	Ben-Tal & Teboulle (2007) [41]	An old-new concept of convex risk measures: The optimized certainty equivalent	2007	227	Article
7	Salamon P & Nitzan (1981) [86]	Finite time optimizations of a Newton’s law Carnot cycle	1981	217	Article
8	Xiao R et al. (2020) [39]	Exploring the interactive coercing relationship between urbanization and ecosystem service value in the Shanghai Hangzhou Bay Metropolitan Region	2020	155	Article
9	Zhou M et al. (2019) [36]	Evidential reasoning approach with multiple kinds of attributes and entropy-based weight assignment	2019	121	Article
10	Lim & Shanthikumar (2007) [45]	Relative entropy, exponential utility, and robust dynamic pricing	2007	112	Article
11	Bellini & Frittelli (2002) [75]	On the existence of minimax martingale measures	2002	95	Article
12	Carrillo H et al. (2015) [85]	Autonomous robotic exploration using occupancy grid maps and graph SLAM based on Shannon and Rényi Entropy	2015	73	Conference paper
13	Liu H et al. (2017) [89]	Entropy-based consensus clustering for patient stratification	2017	72	Article
14	Boukobza & Tannor (2007) [87]	Three-level systems as amplifiers and attenuators: A thermodynamic analysis	2007	70	Article
15	Vinod (2004) [84]	Ranking mutual funds using unconventional utility theory and stochastic dominance	2004	69	Article

Source: Author’s own research using the Biliometrix tool.

## Data Availability

The raw data supporting the conclusions of this article will be made available by the authors on request.
